# Supplementary material for: PYL1- and PYL8-like ABA Receptors of Nicotiana benthamiana Play a Key Role in ABA Response in Seed and Vegetative Tissue
Source: Cells. 2022 Feb 24;11(5):795. doi: 10.3390/cells11050795 (PMC8909036; doi:10.3390/cells11050795)
Supplement: Supplementary file 1 [file cells-11-00795-s001.zip › Figure S1.pdf]

A

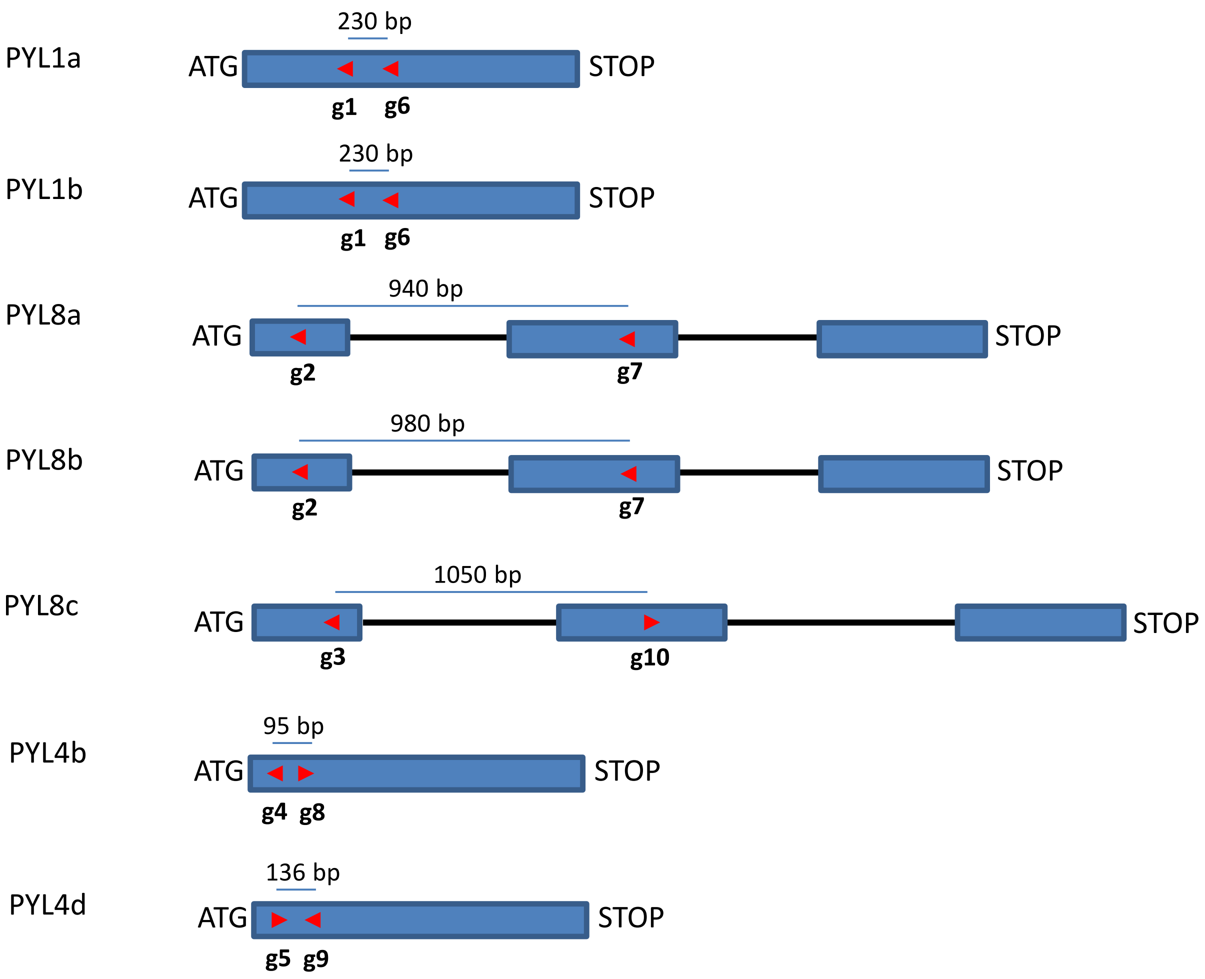

B

Family	Name	Gene ID in database	Gene length (bp)	ORF length (bp)	Protein length (aa)
PYL1 like	PYL1a	NbD001180	678	678	225
PYL1 like	PYL1b	NbD012105	678	678	225
PYL8/9 like	PYL8a	NbD015117	1944	528	175
PYL8/9 like	PYL8b	NbD008843	2020	528	175
PYL8/9 like	PYL8c	NbD004872	3126	558	185
PYL4/5 like	PYL4b	NbD014593	642	642	213
PYL4/5 like	PYL4d	NbE03056248	636	636	212

Figure S1. Target gene sequences and deduced protein size of the gene products. (**A**) Genomic structure of the 7 ABA receptor genes targeted in this study, indicating the gRNAs corresponding to each gene. (**B**) Nomenclature of the genes according to www.nbenth.com database. Gene identity (ID), open reading frame (ORF) length from ATG to stop codon and protein length are indicated.
