# Supplementary material for: PYL1- and PYL8-like ABA Receptors of Nicotiana benthamiana Play a Key Role in ABA Response in Seed and Vegetative Tissue
Source: Cells. 2022 Feb 24;11(5):795. doi: 10.3390/cells11050795 (PMC8909036; doi:10.3390/cells11050795)
Supplement: Supplementary file 1 [file cells-11-00795-s001.zip › Figure S2.pdf]

Figure S2. Chromatograms of the indicated gene sequences for wild-type and mutant plants. Sequence analysis reveals the different insertions (+1) or deletions (Δ1, Δ2, Δ4, Δ24) detected in the CRISPR/Cas9-edited alleles of each ABA receptor

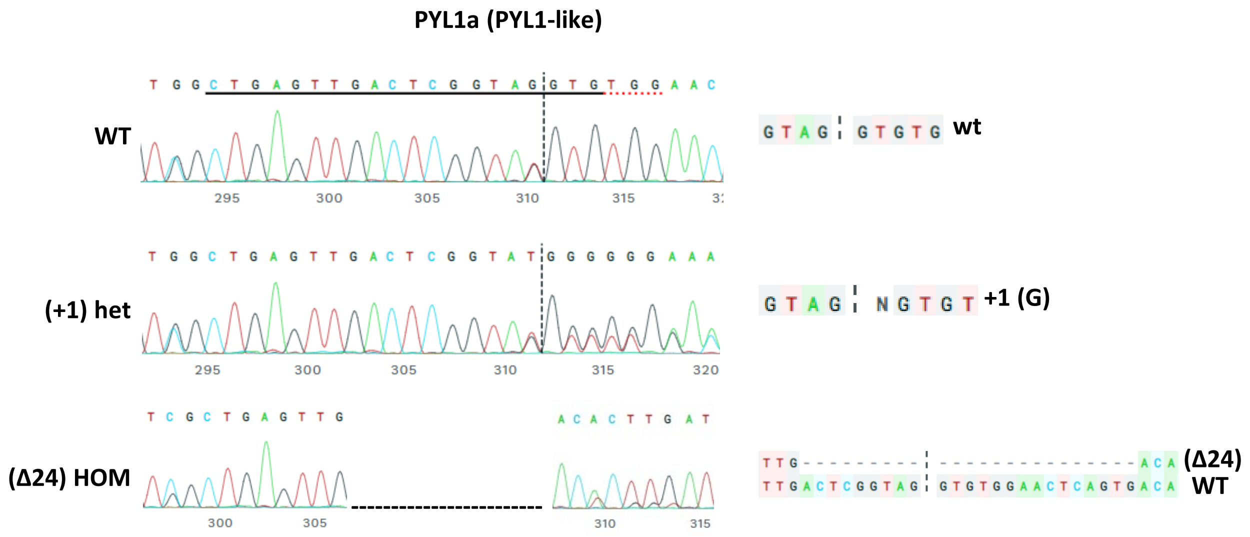

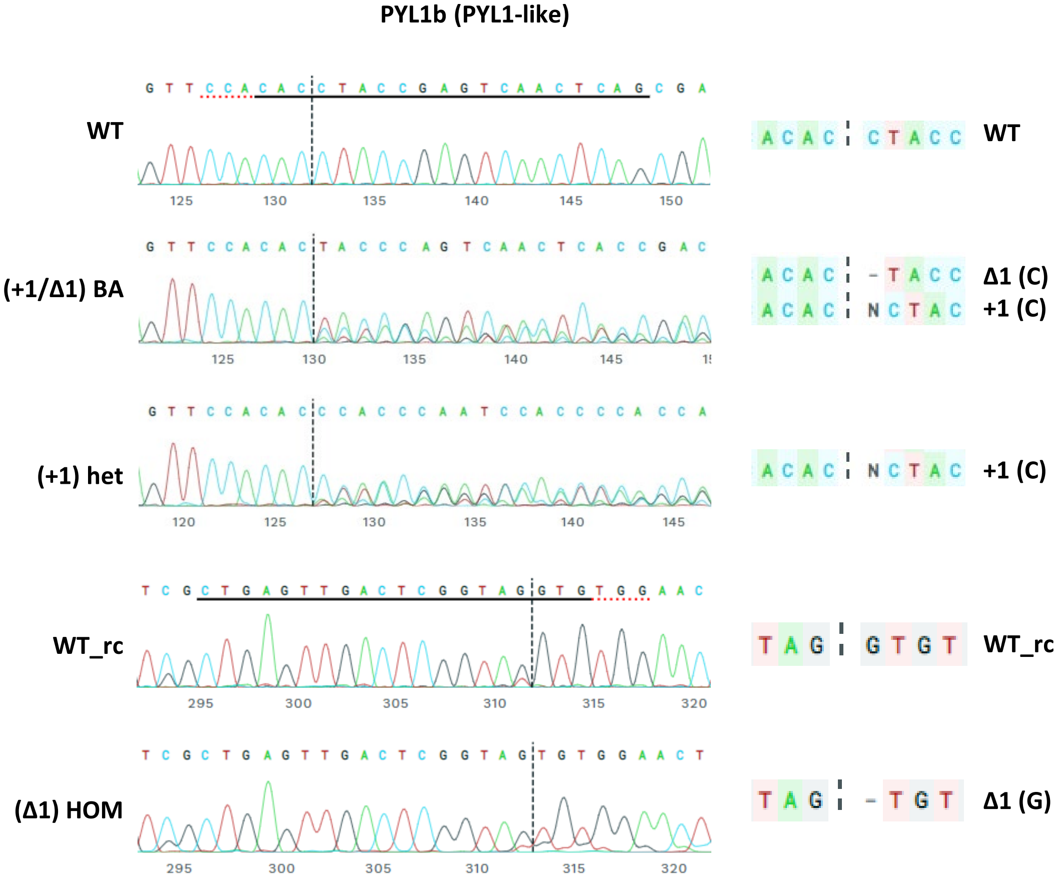

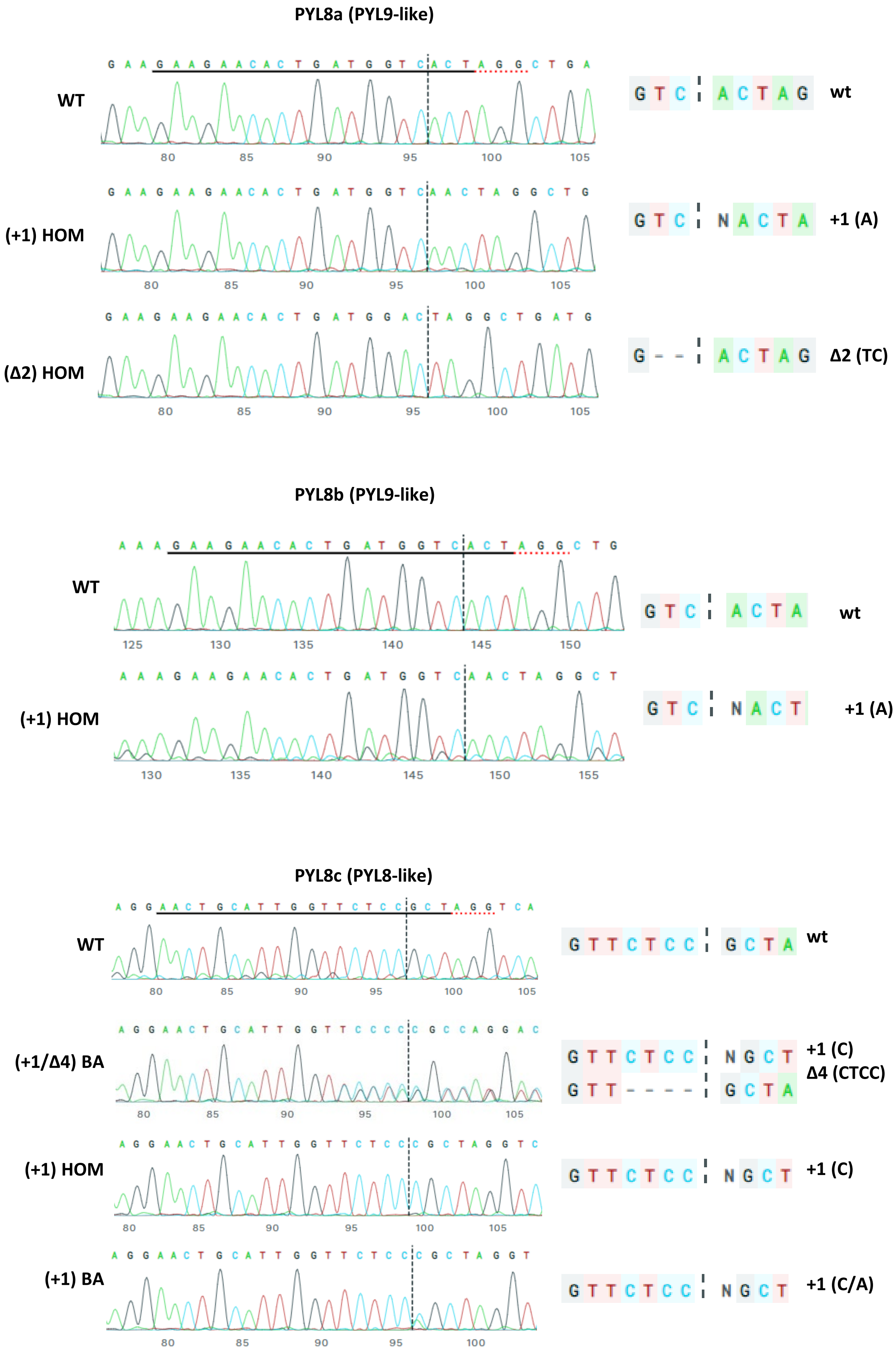
PYL8a (PYL9-like)
WT
G T C A C T A G wt
(+1) HOM
G T C N A C T A +1 (A)
(Δ2) HOM
G - - A C T A G Δ2 (TC)
PYL8b (PYL9-like)
WT
G T C A C T A wt
(+1) HOM
G T C N A C T +1 (A)
PYL8c (PYL8-like)
WT
G T T C T C C G C T A wt
(+1/Δ4) BA
G T T C T C C N G C T +1 (C)
G T T - - - - G C T A Δ4 (CTCC)
(+1) HOM
G T T C T C C N G C T +1 (C)
(+1) BA
G T T C T C C N G C T +1 (C/A)
