# Supplementary figures and images for: PYL1- and PYL8-like ABA Receptors of Nicotiana benthamiana Play a Key Role in ABA Response in Seed and Vegetative Tissue
Source: Cells. 2022 Feb 24;11(5):795. doi: 10.3390/cells11050795 (PMC8909036; doi:10.3390/cells11050795)

A

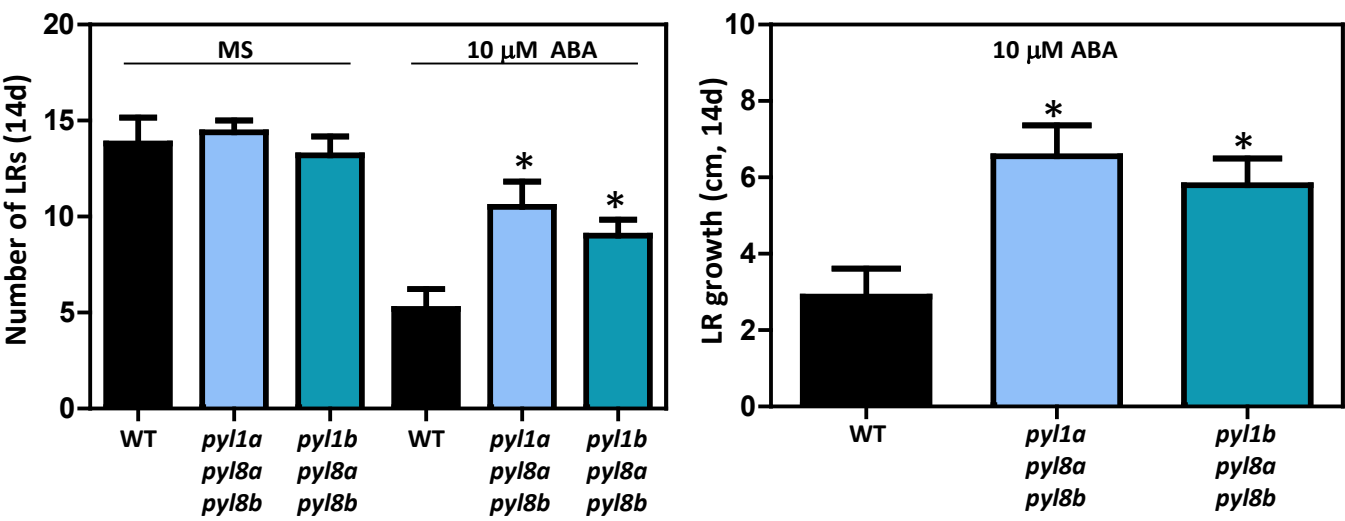

B

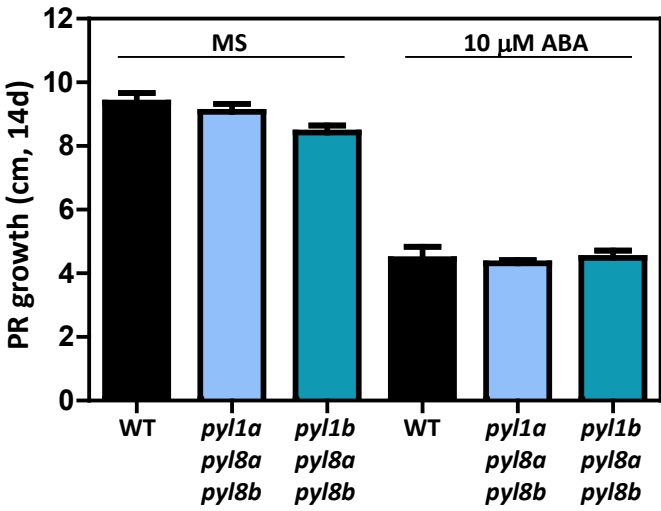

Supplement: Supplementary file 1 [file cells-11-00795-s001.zip › Figure S3.pdf]
